# GPR50 is the mammalian ortholog of Mel1c: Evidence of rapid evolution in mammals

**DOI:** 10.1186/1471-2148-8-105

**Published:** 2008-04-09

**Authors:** Laurence Dufourny, Anthony Levasseur, Martine Migaud, Isabelle Callebaut, Pierre Pontarotti, Benoit Malpaux, Philippe Monget

**Affiliations:** 1 Physiologie de la Reproduction et des Comportements, UMR 6175 INRA-CNRS-Université François Rabelais de Tours-Haras Nationaux, 37380 Nouzilly, France; 2 EA3781: Evolution Biologique – Laboratoire de PhyloGénomique – Université de Provence – Marseille – France; 3 Biologie Structurale, IMPMC, UMR7590, CNRS, Université Paris 6 et 7, 75015 Paris, France

## Abstract

**Background:**

The melatonin receptor subfamily contains three members Mel1a, Mel1b and Mel1c, found in all vertebrates except for Mel1c which is found only in fish, Xenopus species and the chicken. Another receptor, the melatonin related receptor known as GPR50, found exclusively in mammals and later identified as a member of the melatonin receptor subfamily because of its identity to the three melatonin receptors despite its absence of affinity for melatonin. The aim of this study was to describe the evolutionary relationships between GPR50 and the three other members of the melatonin receptor subfamily.

**Results:**

Using an *in silico *approach, we demonstrated that GPR50 is the ortholog of the high affinity Mel1c receptor. It was necessary to also study the synteny of this gene to reach this conclusion because classical mathematical models that estimate orthology and build phylogenetic trees were not sufficient. The receptor has been deeply remodelled through evolution by the mutation of numerous amino acids and by the addition of a long C-terminal tail. These alterations have modified its affinity for melatonin and probably affected its interactions with the other two known melatonin receptors MT1 and MT2 that are encoded by Mel1a and Mel1b genes respectively. Evolutionary studies provided evidence that the GPR50 group evolved under different selective pressure as compared to the orthologous groups Me11 a, b, and c.

**Conclusion:**

This study demonstrated that there are only three members in the melatonin receptor subfamily with one of them (Me11c) undergoing rapid evolution from fishes and birds to mammals. Further studies are necessary to investigate the physiological roles of this receptor.

## Background

The melatonin receptor family belongs to the super family of G protein coupled receptor (GPCR) and contains 3 known subtypes, Mel1a, Mel1b and Mel1c [1-4]. The genes for these receptors contain two exons separated by a large intron [2,3]. The Mel1a gene is found on chromosome 4 in the chicken and human, Mel1c is also found on chromosome 4 in chicken (it is not found in the human), while Mel1b is encoded on chromosome 1 in the chicken and on chromosome 11 in the human. These 3 receptors all bind melatonin with a high affinity (K_D _= 10 to 200 pM) [5,6]. Among them only Mel1a and Mel1b have been cloned and characterized in mammals [7,8] and have been renamed MT1 and MT2 by the International Union of Pharmacology (IUPHAR). In contrast, Mel1c has been found only in fish, the chicken and Xenopus [4]. The three receptors (MT1, MT2 and Mel1c) share about 60% sequence identity and BRET studies showed that MT1 and MT2 receptors could form heterodimers [9].

In eutherian mammals, a previously known orphan receptor GPR50 was identified as a melatonin-related receptor because it has 45% identity with the melatonin receptor family [10]. However GPR50 is encoded by a gene located on the X chromosome and does not bind melatonin [11]. A recent study demonstrated that GPR50 can heterodimerize with MT1 and MT2 receptors [12], leading to a suppression of MT1 but not MT2, affinity for melatonin. Interestingly GPR50 has only been found in eutherian mammals and not in fish or birds.

The aim of this study was to understand the phylogenetic evolution of the melatonin receptor family and more specifically of the Mel1c and GPR50 genes using an *in silico *approach. Studying the phylogenetic tree of the melatonin receptor family and tracking the synteny of genes surrounding Mel1c in several species strongly suggested that the Mel1c gene found in fish and avian species is the ortholog of the eutherian GPR50 gene. This interpretation was further supported by estimation of selection pressure and by a gene structure analysis of the Mel1c and GPR50 genes.

## Results

### Phylogenetic analysis of the melatonin receptors

**Figure 1:**
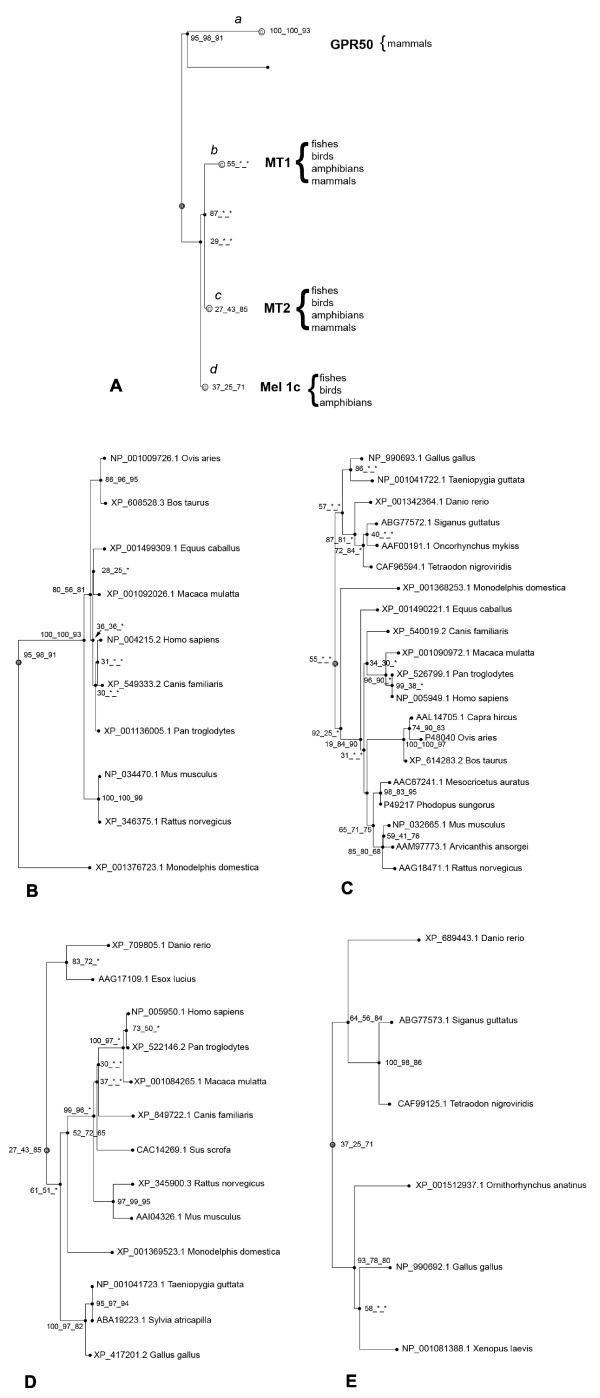
**Phylogenetic analysis of the GPR50/MT1/MT2/Mel1c genes**. (A) Overall phylogenetic tree showing 3 groups of genes: GPR50, MT1/MT2, and Mel1c genes and the animal orders where each branch is expressed. The trees (npl) are the fusion of three phylogenetic trees built based on Neighbour joining, maximum Parsimony and maximum Likelihood (see "Materials and Methods" section for further details). The italic letters correspond to the name given to the branches for the likelihood ratio tests (B) Phylogenetic tree of GPR50 genes. Please note that only mammalian species appear in the tree. (C) Phylogenetic tree of MT1 genes (D) Phylogenetic tree of MT2 genes. (E) Phylogenetic tree of Mel1c genes that do seem to appear only in non mammalian species. Bootstrap values are reported for each npl method.

The phylogenetic tree in Fig. 1A–E, built from the NCBI protein database using chicken *Mel1c *protein as the query, showed that there are four groups of orthologous genes corresponding to *GPR50, MT1, MT2 and Mel1c*. The *GPR50 *gene was only detected in mammalian genomes (Fig. 1B). In contrast, the Mel1c gene was only detected in fish species, Xenopus and chicken genomes confirming previous results [1,4] (Fig. 1E). Noticeably, prototherian and metatherian species appeared in Mel1c (platypus) or GPR50 (oppossum) branches (Fig. 1B, E). Note that several bootstrap values are low suggesting that the robustness of the tree is questionable.

Branch lengths are correlated with the evolutionary rate of the sequence [13] and branch length values were clearly higher for the *GPR50 *orthology group than for the other three groups. These results suggest that sequences from the *GPR50 *group evolved faster than those from *MT1*, *MT2 *or *Mel1c *groups.

Analyses of selection pressure were performed to provide insights into functional constraints applied at sequence level to the GPR50 group. The branch-site model A was applied to test evolutionary shift using the Maximum Likelihood method. The branch leading to the GPR50 group was labelled as the foreground branch and all others as background branches in the phylogenetic tree. Parameter estimates under model A suggested that 71% of sites evolved under purifying selection (ω_0 _= 0) whereas 29% sites were identified under the neutrality assumption. Likelihood Ratio Tests (LTRs) were highly significant with P < 0.0001 (2Δl = 24.7 and df = 2) when the model A was compared to the null model M1a (neutral). Thus, the model did not find evidence for positive selection and that, on average, numerous sites (~25%) evolved without functional constraint.

In order to test if positive selection occurred in different lineages along the phylogeny, others branches (leading to Mel1a, b, c orthologous groups) were labelled as foreground branches. The calculations are summarized in Table 1. All the LRTs gave significant results. Compared to the GPR50 group, the percentage of sites evolving under purifying selection was higher, particularly for the Mel1c group (~95%). Remarkably, the percentage of sites evolving under neutrality for GPR50 was ~28%, contrary to the Mel1a, b, and c groups whose percentages were ~3%. Positive selection was detected in the Mel1a, b, c groups. The Bayes empirical Bayes (BEB) analysis identified 1, 4 and 1 sites respectively, under positive selection along branches leading to Mel1a, b, c, at a probability of >95%. Amino acids under positive selection are reported in Table 1.

**Table 1:** Parameter estimates for the GPR50, mel1 a, b, c under Model A and the effects of codon usage bias on LRTs (n = 29)

Model	*p*	*l*	Parameters estimates	Positively selected sites
M0: one-ratio	103	-15666. 37	ω = 0.0655	
M1a (neutral)	104	-15620.766436	*p*_0 _= 0.967, *p*_1 _= 0.032	
Branch-site model:				
**GPR50:** Model A	106	-15608.39	*p*_0 _= 0.714, *p*_1 _= 0.026 (p_2 _+ *p*_3_) = 0.258 ω_2 _= 1	
**mel1a:** Model A	106	-15613.74	*p*_0 _= 0.883, *p*_1 _= 0.031 (p_2 _+ *p*_3_) = 0.085 ω_2 _= **999**	Site for foreground lineage: 183V
**mel1b:** Model A	106	-15611.52	*p*_0 _= 0.833, *p*_1 _= 0.031 (p_2 _+ *p*_3_) = 0.135 ω_2 _= **999**	98K102Q 147I 227D
**mel1c:** Model A	106	-15617.63	*p*_0 _= 0.955, *p*_1 _= 0.032 (p_2 _+ *p*_3_) = 0.011 ω_2 _= **555.04**	278A

Note: *p *is the number of free parameters for the ω ratios. Parameters indicating positive selection are written in bold. Tests comparing sites-specific model M1a and branch-site model A are significant at 1%, df = 2. Sites potentially under positive selection were identified using GPR50 from *O. aries *according to the Bayes empirical Bayes analyses for Model A (at *P *> 0.95). These sites are shown in Fig. 3.

### Analysis of Mel1c synteny

**Figure 2:**
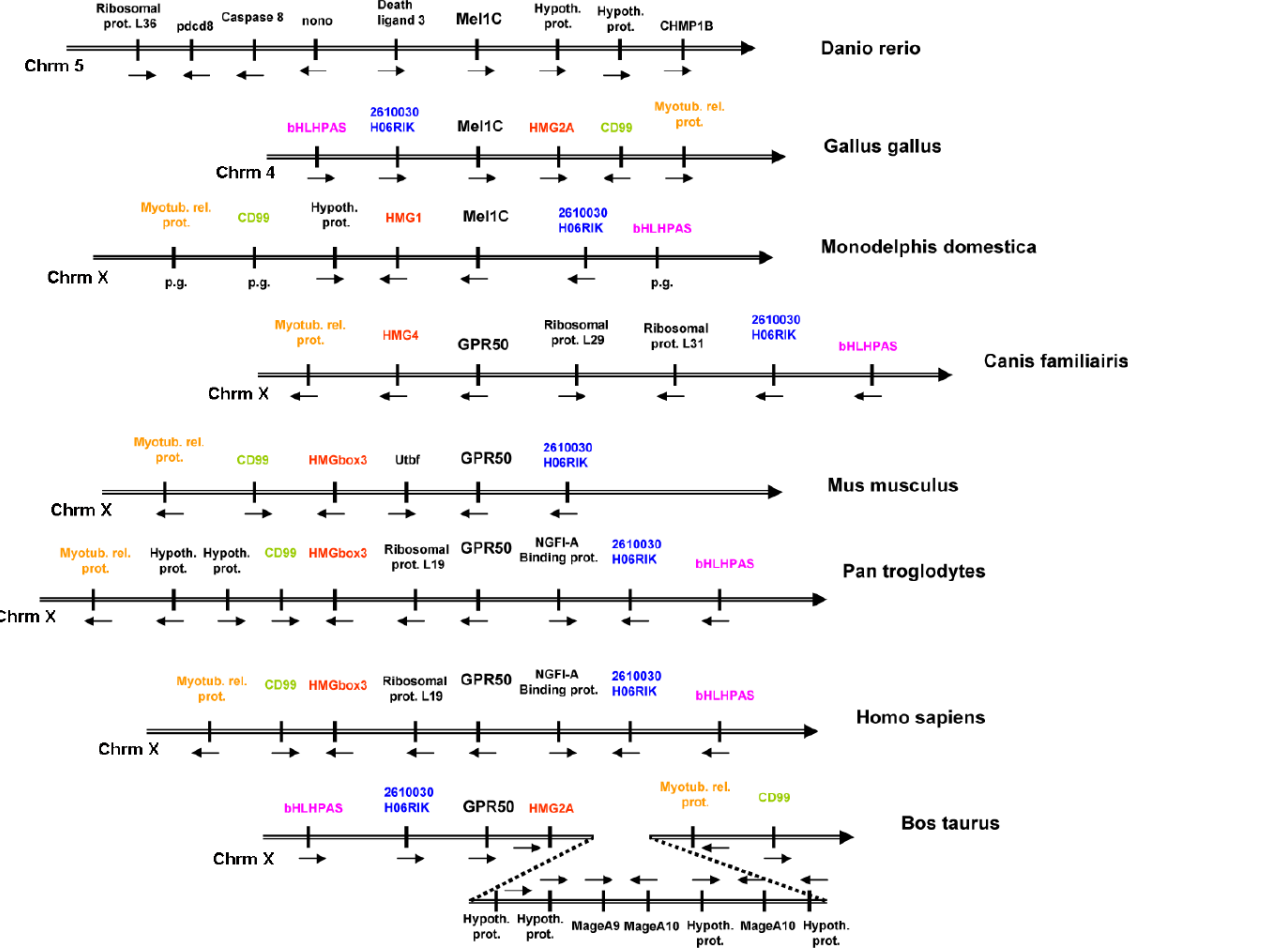
**Synteny of Mel1c/GPR50 genes and neighbours in vertebrate genomes**. Note that genes are found on chromosome 5 in zebra fish and on chromosome 4 in chicken while they are found on chromosome X in other depicted species. Please note that synteny is mostly conserved for bHLHPAS, 2610030H06 RIK, Mel1c, HMG2A, CD99, and myotubularin related protein in opossum and mammalian species despite the integration of new genes coding for hypothetical proteins (opossum, chimpanzee, cow), ribosomal proteins (dog, chimpanzee, man), NGFI-A binding protein (chimpanzee, man), Utbf (mouse) and MAGE (cattle) proteins. It is also of note that several genes surrounding Mel1c in zebra fish (pdcd8, nono, and the two hypothetical proteins) present high identities with genes found on chromosome X in mouse but not in the GPR50 locus (unpublished data). p.d.: predicted gene. Chrm: chromosome.

In order to test whether Mel1c and GPR 50 genes were lost in mammals and in non-mammals species respectively, we carried out a study of the synteny of these genes. At the Mel1c gene locus on chicken chromosome 4 the following group of genes encoding bHLHPAS, Mel1c, HMG2A, CD99 molecule like 2 and myotubularin related protein were displayed (Fig. 2). For clarity, accession numbers of these genes and their orthologs in different species are summarized in Table 2. Interestingly, synteny has been best conserved in mammals where the chromosomal locus containing Me11c is found on the X chromosome (from Ensembl.org website) (Fig. 2). Except for minor changes among species (for example, the insertion of the gene for Ribosomal protein 19 between the genes for GPR50 and HMG in man) this synteny analysis clearly shows that, despite rapid evolution of the coding region of interest, Mel1c evolved into GPR50 in eutherian mammals. A BLAST analysis against all the available mammalian genomes did not reveal any "fossilized" pseudogenes of Mel1c at this locus in any species, strengthening the notion that the Mel1c ancestral gene was not duplicated in mammals to allow the emergence of GPR50 and of a lost Mel1c pseudogene. Interestingly, this rapid evolution was also observed in neighbouring genes, *i.e*. 2610030H06 Rik [see Additional file 1] and HMG2A [see Additional file 2] whose phylogenetic trees show either poor bootstrap values (HMG2A) or an odd organization (2610030H06Rik). This synteny approach represented therefore a powerful tool to elucidate the orthology relationships between fast evolving genes.

**Table 2:** Accession numbers of genes surrounding Mel1c/GPR50. For clarity, the names of proteins used as an entry were based on the protein identification found in chicken.

Protein	bHLHPAS	2610030 H06RIK	Mel1c	HMG2A	CD99 antigen like 2	Myotubularin related protein
*Gallus gallus*	XM_420353.2	NM_001031127.1	NM_205361.1	XM_001235453.1	XM_420355.2	XM_420356.2
*Monodelphis domestica*	NW_001587046.1	XM_001376698.1	XM_001376686.1	XM_001364501.1	NW_001587046.11	NW_001587046.1
*Canis familiaris*	XM_549336.2	XM_538195.2	XM_549333.2	XM_538194.2	N/A	XM_850116.1
*Mus musculus*	N/A	NM_001081356.1	NM_010340.1	NM_008253.3	NM_138309.2	NM_016985.2
*Pan troglodytes*	XR_022949.1	XM_001136167.1	XM_001136005.1	XM_001135755.1	XM_521305.2	XM_521404.2
*Homo sapiens*	NM_173493.1	NM_001017980.1	NM_004224.1	NM_005342.2	NM_134446.2	NM_003828.2
*Bos Taurus*	XM_00125953.1	XM_864689.2	XM_608528.3	NM_001076285.1	XM_614787.3	XM_864632.2

### Sequence alignments between paralogs of the melatonin receptor family, and sequence identity analysis

The percentage of identically aligned amino acids between *MT1*, *MT2*, and *Mel1c/GPR50 *from five species (*Xenopus laevis *(Xl)*, Gallus gallus *(Gg), *Monodelphis domestica *(Md), *Mus musculus *(Mm), *Homo sapiens *(Hs)) are presented in Table 3. The greatest sequence identity among the orthologous genes was observed for MT1 where more than 75% of the amino acid alignment has been conserved if we exclude *Xenopus laevis*.

**Table 3:** Sequence identity analysis

		*Xenopus Laevis*			*Gallus gallus*			*Mododelphis domestica*			*Mus musculus*			*Homo sapiens*		
		MT1	MT2	Mel1c GPR50	MT1	MT2	Mel1c GPR50	MT1	MT2	Mel1c GPR50	MT1	MT2	Mel1c GPR50	MT1	MT2	Mel1c GPR50

*Xenopus laevis*	MT1															
	MT2	*55*														
	Mel1c GPR50	*59*	*71*													
*Gallus gallus*	MT1	**62**	71	70												
	MT2	57	**82**	68	*69*											
	Mel1c GPR50	56	73	**79**	*69*	*72*										
*Monodelphis domestica*	MT1	**57**	65	67	**78**	68	71									
	MT2	38	**50**	64	67	**71**	68	*61*								
	Mel1c GPR50	54	72	**56**	54	53	**57**	*54*	*53*							
*Mus musculus*	MT1	**58**	43	67	**78**	68	66	**75**	64	55						
	MT2	55	**65**	62	60	**65**	61	56	**65**	48	*58*					
	Mel1c GPR50	38	63	**46**	52	51	**46**	48	51	**45**	*53*	*45*				
*Homo sapiens*	MT1	**59**	64	70	**80**	69	70	**77**	61	54	**83**	59	50			
	MT2	54	**70**	64	59	**69**	61	59	**69**	49	61	**82**	48	*61*		
	Mel1c GPR50	39	44	**49**	50	52	**52**	49	48	**47**	52	47	**71**	*51*	*50*	

Percentages of amino acid identity between MT1, MT2, and Mel1c/GPR50 sequences from five species, *Xenopus laevis, Gallus gallus, Monodelphis domestica*, *Mus Musculus*, and *Homo sapiens *All the sequences were compared with a Smith-Waterman local alignment software [57]. All the results ranged from 38% (*Xl*-MT1 *vs Mm*-GPR50) to 83% (*Mm-*MT1 *vs Hs*-MT1) of identity. The percentage of identity between protein sequences of paralogous genes ranged from 50% (*Hs*-MT2 *vs Hs*-GPR50) to 72% (*Gg*-MT2 *vs Gg*-Mel1c). The percentage of identity between protein sequences of orthologous genes ranged from 45% (*Md-*GPR50 *vs Mm*-GPR50) to 83% (*Mm-*MT1 *vs Hs*-MT1).

Percentages of identity between sequences encoded by orthologous genes are written in **bold**.

Percentages of identity between sequences encoded by paralogous genes are written in *italic*.

**Figure 3:**
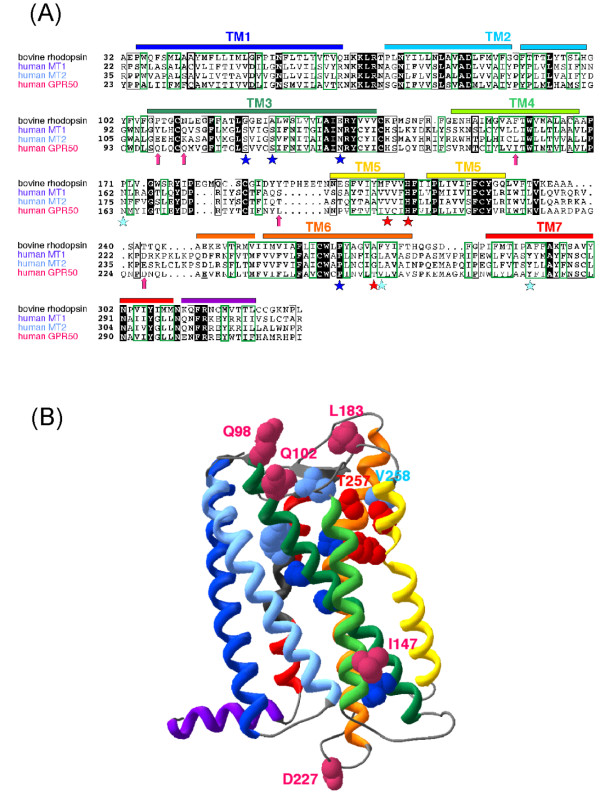
**Sequence alignment of human MT1, MT2 and GPR50 with bovine rhodopsin **(pdb 1F88). Sequence identities are reported white on a black background, whereas sequence similarities are boxed (A). The positions of the transmembrane helices, as observed in the bovine rhodopsin structure, are reported above its sequence. Arrows indicate the positions of the amino acids that, in GPR50, evolved under positive selection. Stars indicate amino acids which have been shown to play a key role for melatonin binding in MT1 (dark blue), MT2 (light blue) or both (red). A ribbon representation of the GPR50 3D structure model is represented (B), with transmembrane helices colored according to the sequence alignment. Amino acids evolving under positive selection and amino acids important for melatonin binding in MT1/MT2 are shown according to the colors reported in the sequence alignment.

Mutagenesis studies performed either on the human [14-17] or ovine MT1 receptor [18] and studies using human chimeric GPR50/MT1 receptor constructs [19,20] have shown several highly conserved residues in transmembrane helices that are critical for ligand binding especially those in transmembrane helix III (TMIII: S110, G258) (Fig. 3). In addition, mutation of the N124 within the specific NRY signature of the melatonin receptor group, located just downstream of TMIII dramatically impairs receptor function (binding affinity, control of cAMP level and regulation of ion channel activity [21]). In the same way, key amino-acid residues in the ligand binding pocket of the MT2 receptor are located in transmembrane helices IV (N175), V (V204, V208), VI (G271, L272) and VII (Y298) ([22,23]; Fig. 3). These residues are extremely conserved among species and receptor subtypes. It is worth noting that some key amino acids for melatonin binding that are found in helix VI of the MT1 receptor (G258) and of the MT2 (G271, L272) are substituted by T257 and V258 respectively in human GPR50 (Fig. 3A)

### Structural evolution of Mel1c into GPR50

**Figure 4:**
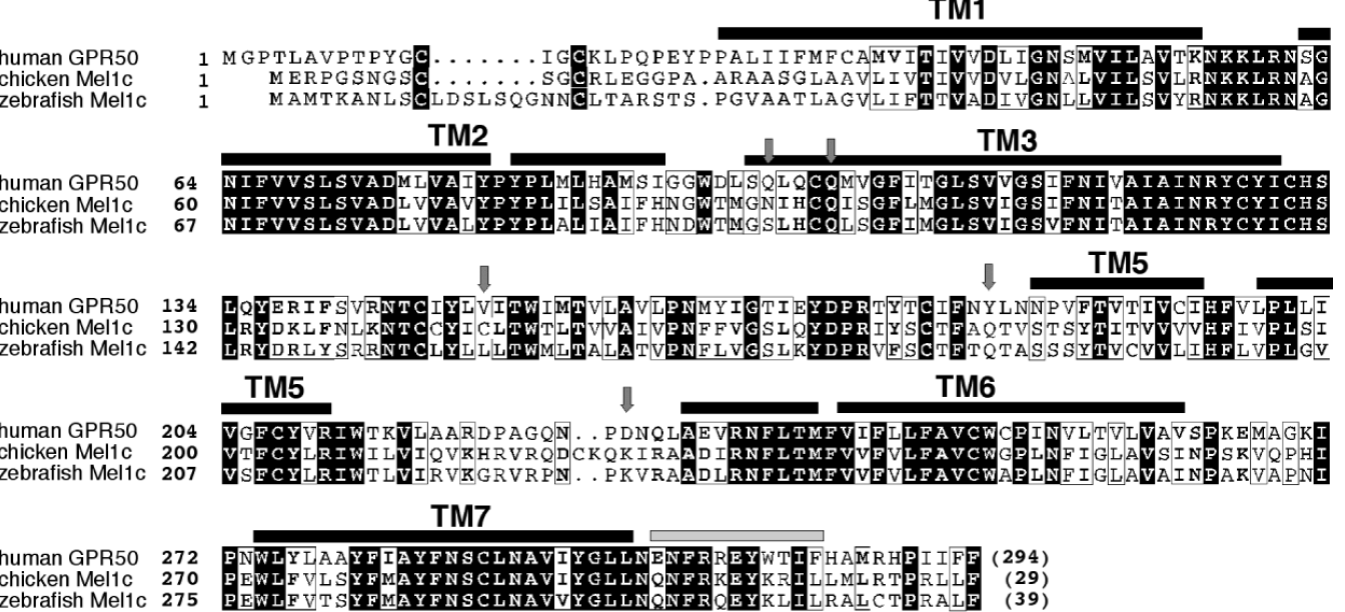
**Sequence alignment of chicken Mel1c, zebra fish Mel1c and human GPR50**. Sequence identities are reported white on a black background, whereas sequence similarities are boxed. The positions of the transmembrane helices are reported above its sequence. Arrows indicate the positions of the amino acids that, in GPR50, evolved under positive selection.

Sequence alignments of the orthologous genes Mel1c and GPR50 (Fig. 4) reveal the addition of a long C terminal domain in the GPR50 receptor. As a consequence, the largest discrepancies between the sequence alignment of amino acids was observed for the Mel1c and GPR50 orthologs where the sequence identity ranged from 45% to 79% (Table 3). This led us to compare the gene structure of the Me11c and GPR50 receptors.

**Figure 5:**
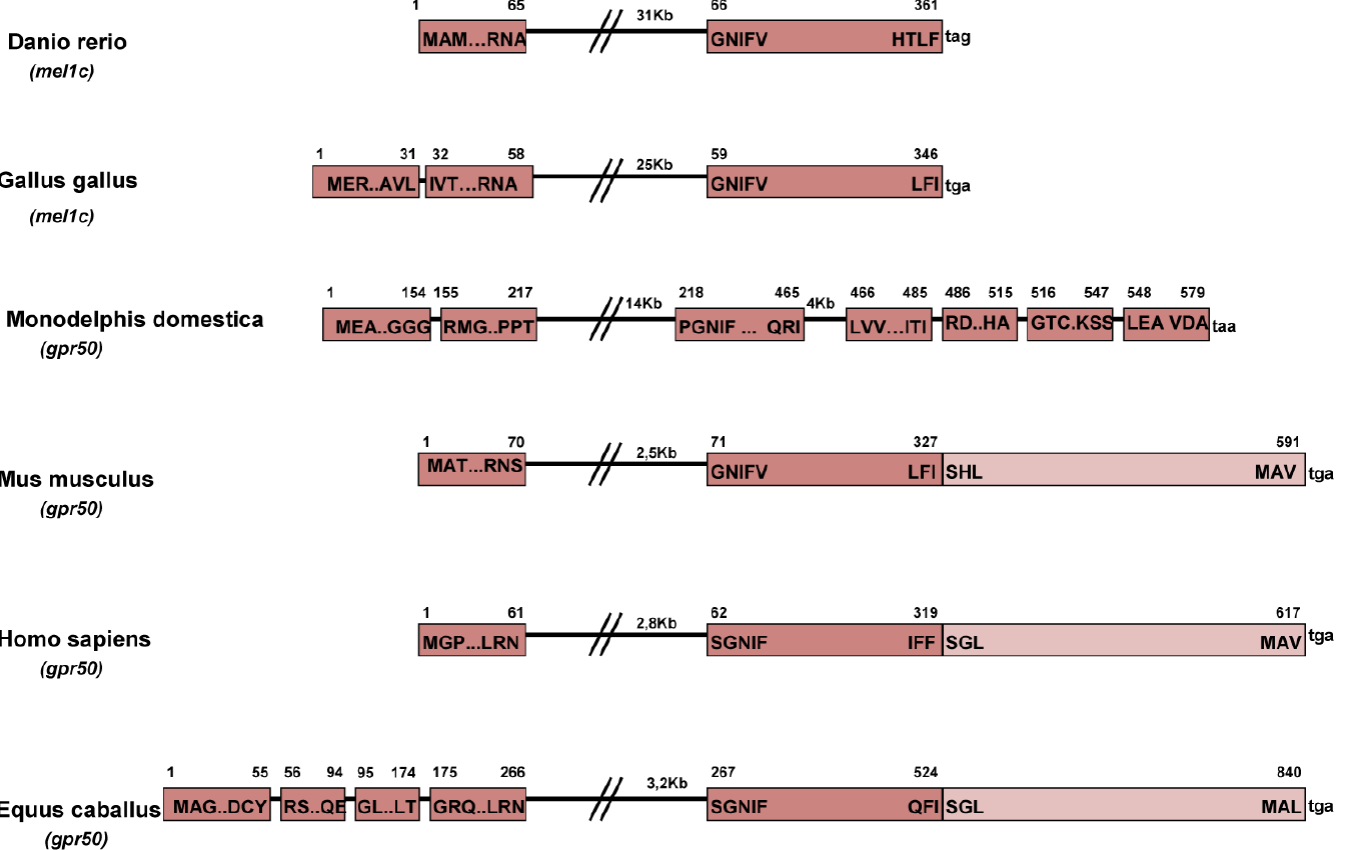
**Schematic diagram of the Mel1c/GPR50 gene organization in zebrafish, chicken, opossum, mouse, man, and horse**. The stop codon following the second exon in zebrafish and chicken is replaced by the insertion of a protein fragment reminiscent of a DNA directed RNA polymerase II in mammals (light color).

**Figure 6:**
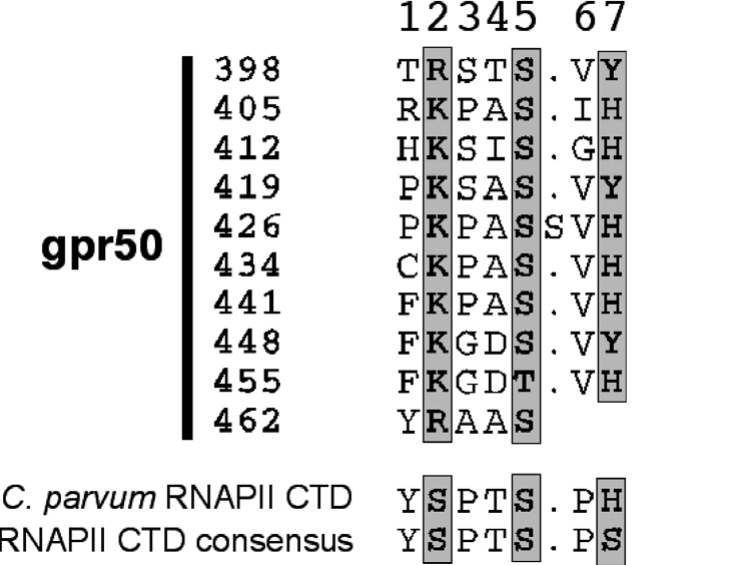
**Alignment of the repeated heptad found in the C-terminal extension of human GPR50 and comparison with the repeated heptad observed in the C-terminal domain (CTD) of RNA polymerase II (RNAPII)**. The three positions (2, 5 and 7) occupied by phosphorylable serine residues in RNAPII CTD are boxed.

Study of Mel1c structures in several species using BLAT software revealed a common gene organization in the zebra fish and the chicken where Mel1c was coded by 2 exons (Fig. 5). The structure of the GPR50 gene was very similar in mouse and man where it also contained 2 exons, but the first exon is segmented into 4 smaller exons in the horse. In contrast, the GPR50 gene in the opossum was made up of 7 smaller exons. The C-terminal fragment in 3' position in eutherian mammals replaces the stop codon found in the chicken and zebra fish; the fragment starts at the 3' end with a SxL amino acid sequence, S being amino acid 328 in the mouse, 320 in man and 525 in the horse (Fig. 5). Analysis of this extension to the C-terminus of the receptor using a bidimensionnal Hydrophobic Cluster Analysis (HCA; hydrophobic residues gathered into clusters, typical of regular secondary structures) identified a repeated sequence between amino acids 398 and 466 (Fig. 6). This repeated sequence is organized around a degenerated heptapeptide. The first and last positions of the heptad are generally occupied by an aromatic amino acid, the sixth position by an aliphatic, hydrophobic amino acid, and the second and fifth positions are occupied by a basic amino acid (generally K) and by a hydroxyl amino acid (generally S). One of the ten repeats has a single amino acid insertion (S) between the fifth and sixth positions. This repeat heptad is reminiscent of the C-terminal repeat domain (CTD) of RNA polymerase II (RNAPII), with which it well aligned (Fig. 6). The RNAPII CTD also has an unusual extension, outside the catalytic core of the largest subunit of the enzyme, that serves as a flexible binding scaffold for numerous factors that regulate transcription-related events (for reviews, see [24,25]). The binding of factors to the RNAPII CTD is determined by the pattern of phosphorylation, which principally occurs at Ser2 and Ser5 of the repeat. Worth noting, is that the second and fifth positions in GPR50 repeats include amino acids that are highly conserved. A serine is also highly conserved in the fifth position, as for RNAPII CTD, whereas a basic amino acid (K or R) is invariably conserved in the second position instead of a serine. This conserved pattern in GPR50 together with its similarity to RNAPII CTD, suggests that GPR50 repeats might also constitute a flexible scaffold for the binding of partner(s) that recognize specific phosphorylation sites. The GPR50 heptad repeat is followed by a ~100 amino acid domain. And from HCA analysis the domain is predicted to be structured. However, this domain is unusually rich in serine and threonine residues and many other repeated sequences (e.g. a SH dipeptide is repeated five times at non regular intervals). These sequences are probably in specific structures and functions.

## Discussion

Using an *in silico *approach, we have demonstrated in this study, that Mel1c, the gene for a high affinity melatonin binding receptor found in the chicken and in Xenopus, rapidly evolved into GPR50 gene in eutherian mammals. The GPR50 gene encodes a receptor that does not bind melatonin but affects the interaction of this hormone with its cognate MT1 receptor after dimerization.

Analysis of the phylogenetic tree of the melatonin receptor family suggested that Mel1c is not present in mammals. Two evolutionary hypotheses are possible. The first, is duplication of the Mel1c/GPR50 ancestral gene before the emergence of vertebrate species. One of these genes evolved into the Mel1c gene of fish species, Xenopus and the chicken, the GPR50 gene being lost in these species, while in mammals, the ancestral gene evolved into GPR50 and the Mel1c gene was lost. This hypothesis implies the existence of a Mel1c pseudogene in mammals and a GPR50 pseudogene in fishes, Xenopus and chicken. A similar pathway exists for the zona pellucida gene family [26] where the ZPAX and ZPD genes have been lost in mammals. However, careful analysis of BLAST data against all the genomes studied failed to find any evidence for "fossilized" genes for GPR50 or Mel1c for example, a residual exon with a stop codon or a deletion. Thus it is unlikely that Mel1c/GPR50 evolved as a consequence of gene duplication. The second more likely hypothesis is that the Mel1c gene evolved rapidly into GPR50 gene in mammals by the mutation of several critical amino acids and by the addition of a C-terminal sequence. A condition for this hypothesis to be true is that the Mel1c and GPR50 genes are surrounded by the same genes, in the syntenic genomic regions of the chicken, opossum and mammalian genomes. This conserved synteny has been clearly highlighted in our results. In this regard, the Mel1c/GPR50 gene is located close to a break in the synteny of the chromosome. Breaks of synteny zones are associated with regions of chromosomal instability in rodents [27]. Navarro and Barton (2003) have also demonstrated that the *Ka/Ks *ratio is higher for genes located on chromosomes that underwent structural rearrangements between the human and the chimpanzee compared with colinear chromosomes [28]. It is therefore possible that a link exists between the rapid evolution of Mel1c into GPR50 gene in mammals and its close vicinity to a site of structural rearrangement in the chromosome.

Considering the rapid evolutionary process of Mel1c into GPR50, two interesting features are worth highlighting. The first is the loss of affinity of GPR50 for melatonin. It is widely assumed that G protein-coupled receptors and among them, the melatonin receptors, share the same structure as rhodopsin in which predicted critical residues for ligand binding to the appropriate binding pocket are located in the transmembrane regions [29]. Mutagenesis studies performed either on human MT1 or MT2 receptors have shown that several transmembrane amino-acid are critical for the binding of melatonin. Among these, the critical amino acids for melatonin binding G258 in helix VI of MT1 and G271 and L272 of MT2 are replaced by T257 and V258 in human GPR50. Interestingly, these amino acid substitutions have probably evolved under conditions of neutrality strengthening the neutral evolution theory of Kimura [30], even if at present, there no available tools to confirm this theory. Our results show that five other separate sites (one, four and one amino acids for MT1, MT2 and Mel1c respectively) also underwent rapid evolution under positive selection. One of these amino acids (L183), in the second extracellular loop of the GPR50, has replaced S or T in all melatonin receptor subtypes of all species except in *Esox lucius *MT2 (A197). Whether or not these amino acid substitutions have actually led to the loss of affinity of the GPR50 for melatonin may be confirmed by site-directed mutagenesis. To explain the loss of affinity of GPR50 for melatonin, we observed that the amino substituted under positive selection were surprisingly not the ones identified as being important for high affinity binding. The 3D structural model reveals that both types of sites (positively selected sites and sites important for melatonin binding), although distinct, are close to each other leading to potential impairment of ligand binding. The GPR50, 3D model also shows that three of the sites under positive selection are located at the external membrane surface of the receptor. At this position they might be able to "gate" the receptor ligand pocket and therefore contribute to loss of affinity of GPR50 for melatonin.

The second intriguing feature of Mel1c/GPR50 evolution is the addition of the long C-terminal tail. This region may have also contributed to the functional differences between GPR50 and Mel1c. This CTD shows no signs of rapid evolution because it is well conserved among mammals. It is of note that the opossum GPR50 gene contained four more exons coding for the CTD of this receptor than eutherian mammals. However, PSI-BLAST did not reveal any significant homology of the last 4 exons with a known protein suggesting that this CTD appeared after the divergence of non-eutherian and eutherian mammals. The physiological function of this CTD remains to be established.

Despite the functional difference between GPR50 and Mel1c no positive selection was detected suggesting that functional shifts not always correlated with positive selection or that the model used for detecting positive selection is unable to discriminate sufficiently. However, the percentage of sites in the GPR50 group (~28%) that on average, evolve under neutrality allows us to hypothesize that relaxed selection is linked to functional change. This percentage for the branch leading to the Mel1a, b, c groups is ~3% suggesting that the melatonin receptor family has a divergent evolutionary history. Currently, only a few examples of relaxed or positive selection have been related to functional shifts but the role of the environment in gene evolution must be statistically examined before any such claims can be substantiated [31]. Key insights into understanding the role of the environment on the evolution of GPR50 would be gained by pinpointing the selective advantage conferred in mammals by the change. Determining the physiological roles of this receptor is therefore critical to the testing of this hypothesis.

The rapid evolution of Mel1c into GPR50 is associated with a change in the physiological role of the the pineal gland, from a directly photosensitive organ with its own photoreceptors in most non-mammalian vertebrates into an indirectly photosensitive neuroendocrine gland in mammals [32]. Moreover, there is a difference between fishes and mammals in the cellular pathways inducing genes in the pineal gland. One is the recently described orphan nuclear receptor Rev-erb α [33]. This gene is regulated by orthodenticle homeobox 5 (Otx5) through a Pineal Expression Related Element (PERE) that is found in fish species and Xenopus but not in mammals [33]. Nishio and co-workers suggest that Rev-erb α gene expression in the pineal underwent a major functional shift in mammals. Interestingly, it was previously reported that Otx5 family of genes undergoes rapid evolution in mammalian lineages where they are known as Crx genes, with a restricted distribution compared to Otx genes [34]. Taken together with our data, these results suggest that some components of the photoresponsive network, from the genes expressed in the pineal gland to melatonin receptors in the hypothalamus, underwent particularly rapid evolution in vertebrates. However the precise significance of this evolution still needs to be clarified.

The functional significance Mel1c and its evolution into into GPR50 are unclear partly because of the lack of knowledge concerning the role of Mel1c receptors in the intracellular transduction of the melatonin signal. Moreover, the physiological regulation [35,36] and function [12,36-38] of GPR50 receptors remain largely unknown despite a recent paper indicating an altered metabolic phenotype of GPR50 knockout mice [36]. Interestingly, Mel1c and GPR50 receptors do not share the same distributions in the brain. In the chicken, Mel1c is widely spread in the brain [4], while in mammals GPR50 has been observed in hypothalamo-pituitary regions, for example dorsomedial hypothalamus in the rodent [36,39] and the pars tuberalis in the human and the sheep [10,11], a noticeable exception is the ependymal cell layer of the third ventricle where strong GPR50 expression is seen in all species examined to date [35,36]. This altered pattern of expression and the loss of affinity for melatonin is probably not a neutral physiological event with respect to melatonin signalling. Melatonin is secreted throughout the period of darkness and the duration of its secretion mimics daylength during the year [40]. This signal constitutes a chemical transduction of the time of the year and is a critical factor for the regulation of seasonal functions such as moulting, hibernation and reproduction in mammals (for reviews see: [41,42]). It is intriguing that GPR50 is present mainly in the ependymal cell layer of the third ventricle [11,35,43], where cerebrospinal fluid concentrations of melatonin are up to 20 times higher than in blood [44]. In addition, levels of GPR50 in ependymal cells of Siberian hamsters are lower under photoperiodic conditions that mimic short days [35]. A recently published *in vitro *study reported decreased function of the MT1 receptor after heterodimerization with GPR50 because of an interaction of the C-terminal tail of GPR50 with regulatory proteins of MT1 receptors [12]. It is thus tempting to speculate that the photoperiodic modulation of GPR50 regulates the function of the MT1 receptor by altering its affinity for melatonin, and hence modulates physiological responses elicited by this receptor. A critical test of such a hypothesis is to determine if MT1 receptors are in the ependymal cell layer of the third ventricle. Presently this is difficult because there is not a suitable MT1 receptor antibody and because of low levels of MT1 mRNA [42].

## Conclusion

In conclusion, this work has shown that the methodology allowing the building of phylogenetic trees may not be sufficient to define the orthological relationships among genes that evolve rapidly, and that studying synteny is often necessary to decipher the relationships among genes of a family. When applied to the family of melatonin receptor genes, this approach allowed us to demonstrate that the high affinity melatonin receptor Mel1c found in non mammalian species is present in the genomes of mammalian species where it has been named GPR50. This receptor has been extensively remodelled through evolution by the mutation of numerous critical amino acids and by the addition of a long C-terminal tail. These alterations have modified the affinity of GPR50 for melatonin and probably affected its interactions with the two functional melatonin receptors, MT1 and MT2, in mammals. Further studies are required to determine the physiological roles of the GPR50 receptor.

## Methods

### Phylogenetic analysis

We performed the phylogenetic analysis using the phylogenomic analysis pipeline available in FIGENIX platform [45,46]. The FIGENIX platform retrieved sequences, provided multiple sequence alignments, phylogenetic reconstruction and deduced orthology and paralogy relationships (for a detailed description of pipelines and models used, see [46]). The chicken *Mel1c *protein sequence (346 aa) was extracted from NCBI (accession no. NP_990692.1) and entered in the phylogenomic inference task, which was run with the default parameters and with Ensembl or NCBI protein database. We also built trees of chicken Mel1c flanking protein sequences: 2610030H06 Rik (Accession number NM_001031127.1) and HMG2A (Accession number XM_001235453.1). We chose the NJ topology for the figures. The trees (npl) are the fusion of three phylogenetic trees built based on Neighbour joining [47], maximum Parsimony and maximum Likelihood [48]. The Dayhoff PAM matrix [49] provided the distance matrix for the NJ method. The evolutionary distance separating sequences is defined as the number of mutational events per site underlying the evolutionary history separating the sequences. Thus, evolutionary relations among sequences are represented by a tree structure where branch length represents the evolutionary distance [13]. In Fig 1, and in additional files 1 and 2, for each node, bootstrap values are reported for each npl method. Bootstrapping was carried out with 1000 replications.

### Evolutionary shift analysis

The protein sequences were aligned using Clustal W [50]. Correspondence between protein alignment and each DNA sequence was established using the Wise2 software package followed by manual adjustments [51]. The final alignment contained 783 codons and 52 aligned sequences: The codeml program of the PAML (Phylogenetic Analysis by Maximum Likelihood [52]) 3.15 software package was applied to test evolutionary shift, PAML uses a Maximum Likelihood algorithm to assign likelihood scores to different models for selection. We first used the model A that enables ω (= *d*N/*d*S) to vary both between sites and between lineages, and was implemented in the maximum likelihood framework [53]. Branches *a, b, c *and *d *were independently labeled as foreground branches, and all remaining branches were labeled as background branches (see Fig. 1). This model was then used to construct likelihood ratio tests (LRTs) by comparison with the null model (site model M1a neutral).

### Analysis of Mel1c synteny

We examined the synteny of genes flanking Me11c on chicken chromosome 4: bHLH-PAS (XM_420353.2), 2610030H06 (NM_001031127.1), HMG2A (XM_001235453.1), CD99 molecule like 2 (XM_420355.2) and myotubularin related protein (XM_420356.2). These genes are found on chromosome 4 whose synteny in mammals is found on chromosome X [54]. Using the "TBLASTN" software [55], proteins were related to sequences of the genome of the opossum (*Monodelphis domestica*), dog (*Canis familiaris*), mouse (*Mus musculus*), chimpanzee (*Pan troglodytes*), human (*Homo sapiens*) and cattle (*Bos taurus*) (Fig. 2). For clarity, accession numbers of species examined for different gene orthologs are summarized in Table 2. For comparison, the synteny of genes flanking Mel1c found on zebra fish (*Danio rerio*) chromosome 5 were also added (Fig. 2). Using the TBLASTN software, we found several genes surrounding Me11c in that species (pdcd8, nono, and the two hypothetical proteins) with a high percentage identity with genes on chromosome X of the mouse but at different loci than GPR50 (data not shown).

### Multiple sequence alignments (MT1, MT2, Mel1c and GPR50)

Multiple alignments of the amino-acid sequences of Mel1a, Mel1b and Mel1c/GPR50 were performed using the Clustal W software available at the EMBL-European Bioinformatics institutes web site [56] (Figs. 3 and 4).

### Sequence identity analysis

Amino acid sequences of Mel1a, Mel1b and Mel1c/GPR50 for *Xenopus laevis, Gallus gallus, Monodelphis domestica, Mus Musculus and Homo sapiens *were aligned by pairs using the Smith-Watermann local alignment (EMBOSS) software [57]. The program compares protein sequences and calculates the statistical significance of matches. For each alignment, we focused on the percentage of amino acid identity (Table 3).

### Gene structure analysis of GPR50

Protein sequences coding for Mel1c in chicken and zebra fish and for GPR50 in opossum, mouse, horse and man were run through the "BLAT" software [58] to deduce the gene structure (Fig. 5)

### Analysis of the C-terminal extension of GPR50

The sequence of the C-terminal extension of human GPR50 does not share any obvious similarity with other proteins available in database search sensitive programs such as PSI-BLAST [59]. The bi-dimensional method of sequence analysis, called Hydrophobic Cluster Analysis [60,61], which efficiently combines analysis of the 1D and 2D structures, was used to explore further the GRP50 C-terminal extension. This led to the identification of repeated sequences, which are described in the Results section (Fig. 6)

### GPR50 homology modelling

A model of the three-dimensional structure of the GPR50 transmembrane domain was obtained using the high resolution crystal structure of bovine rhodopsin (pdb 1F88) as a template. The multiple alignment of bovine rhodopsin with MT1, MT2 and GPR50, shown in Fig. 3, was performed using MAFFT [62] and refined using Hydrophobic Cluster Analysis (HCA; [60]). This alignment is similar to that reported by Rivara *et al*. for MT1 and MT2 receptor models [63]. The three-dimensional models (Fig. 3) were generated using MODELLER [64] and their stereochemical quality checked using PROCHECK [65].

## Authors' contributions

The synteny study was performed by LD who also, built the phylogenetic trees, and drafted the manuscript. The evolutionary shift analysis was carried out by AL who also helped draft the manuscript, MM participated in the sequence alignments and helped draft the manuscript, IC performed the 3D protein study, the CTD analysis and helped draft the manuscript. The evolutionary shift analysis was carried out in part, by PP who also participated in the analysis of the phylogenetic trees, BM was involved in the design of the study and helped draft the discussion and PM was involved in the design and coordination of the study and helped draft the manuscript. All authors read and approved the final manuscript.

## Supplementary Material

Additional file 1 Phylogenetic tree for 2610030H06Rik gene. The figure provided shows the odd organization of species within the tree (for example, chicken associated in the same branch than opossum), which suggests a fast evolution of the 2610030H06Rik gene. Click here for file

Additional file 2 Phylogenetic tree for HMG2A gene. The figure provided highlights the poor bootstrap values in the tree which suggests a fast evolution of HMG2A gene. Click here for file

## References

[B1] Ebisawa T Karne S Lerner MR Reppert SM Expression cloning of a high-affinity melatonin receptor from Xenopus dermal melanophores Proc Natl Acad Sci U S A 1994 91 13 6133 6137 10.1073/pnas.91.13.6133 7517042 PMC44152

[B2] Reppert SM Weaver DR Ebisawa T Cloning and characterization of a mammalian melatonin receptor that mediates reproductive and circadian responses Neuron 1994 13 5 1177 1185 10.1016/0896-6273(94)90055-8 7946354

[B3] Roca AL Godson C Weaver DR Reppert SM Structure, characterization, and expression of the gene encoding the mouse Mel1a melatonin receptor Endocrinology 1996 137 8 3469 3477 10.1210/en.137.8.3469 8754776

[B4] Reppert SM Weaver DR Cassone VM Godson C Kolakowski LF Jr. Melatonin receptors are for the birds: molecular analysis of two receptor subtypes differentially expressed in chick brain Neuron 1995 15 5 1003 1015 10.1016/0896-6273(95)90090-X 7576645

[B5] Audinot V Mailliet F Lahaye-Brasseur C Bonnaud A Le Gall A Amosse C Dromaint S Rodriguez M Nagel N Galizzi JP Malpaux B Guillaumet G Lesieur D Lefoulon F Renard P Delagrange P Boutin JA New selective ligands of human cloned melatonin MT1 and MT2 receptors Naunyn Schmiedebergs Arch Pharmacol 2003 367 6 553 561 10.1007/s00210-003-0751-2 12764576

[B6] Nonno R Lucini V Spadoni G Pannacci M Croce A Esposti D Balsamini C Tarzia G Fraschini F Stankov BM A new melatonin receptor ligand with mt1-agonist and MT2-antagonist properties J Pineal Res 2000 29 4 234 240 10.1034/j.1600-0633.2002.290406.x 11068946

[B7] Dubocovich ML Masana MI Iacob S Sauri DM Melatonin receptor antagonists that differentiate between the human Mel1a and Mel1b recombinant subtypes are used to assess the pharmacological profile of the rabbit retina ML1 presynaptic heteroreceptor Naunyn Schmiedebergs Arch Pharmacol 1997 355 3 365 375 10.1007/PL00004956 9089668

[B8] Ting KN Blaylock NA Sugden D Delagrange P Scalbert E Wilson VG Molecular and pharmacological evidence for MT1 melatonin receptor subtype in the tail artery of juvenile Wistar rats Br J Pharmacol 1999 127 4 987 995 10.1038/sj.bjp.0702612 10433507 PMC1566088

[B9] Ayoub MA Couturier C Lucas-Meunier E Angers S Fossier P Bouvier M Jockers R Monitoring of ligand-independent dimerization and ligand-induced conformational changes of melatonin receptors in living cells by bioluminescence resonance energy transfer J Biol Chem 2002 277 24 21522 21528 10.1074/jbc.M200729200 11940583

[B10] Reppert SM Weaver DR Ebisawa T Mahle CD Kolakowski LF Jr. Cloning of a melatonin-related receptor from human pituitary FEBS Lett 1996 386 2-3 219 224 10.1016/0014-5793(96)00437-1 8647286

[B11] Drew JE Barrett P Williams LM Conway S Morgan PJ The ovine melatonin-related receptor: cloning and preliminary distribution and binding studies J Neuroendocrinol 1998 10 9 651 661 10.1046/j.1365-2826.1998.00229.x 9744482

[B12] Levoye A Dam J Ayoub MA Guillaume JL Couturier C Delagrange P Jockers R The orphan GPR50 receptor specifically inhibits MT1 melatonin receptor function through heterodimerization Embo J 2006 25 13 3012 3023 10.1038/sj.emboj.7601193 16778767 PMC1500982

[B13] Nei M Phylogenetic analysis in molecular evolutionary genetics Annu Rev Genet 1996 30 371 403 10.1146/annurev.genet.30.1.371 8982459

[B14] Kokkola T Watson MA White J Dowell S Foord SM Laitinen JT Mutagenesis of human Mel1a melatonin receptor expressed in yeast reveals domains important for receptor function Biochem Biophys Res Commun 1998 249 2 531 536 10.1006/bbrc.1998.9182 9712731

[B15] Conway S Mowat ES Drew JE Barrett P Delagrange P Morgan PJ Serine residues 110 and 114 are required for agonist binding but not antagonist binding to the melatonin MT(1) receptor Biochem Biophys Res Commun 2001 282 5 1229 1236 10.1006/bbrc.2001.4722 11302748

[B16] Gerdin MJ Masana MI Ren D Miller RJ Dubocovich ML Short-term exposure to melatonin differentially affects the functional sensitivity and trafficking of the hMT1 and hMT2 melatonin receptors J Pharmacol Exp Ther 2003 304 3 931 939 10.1124/jpet.102.044990 12604667

[B17] Kokkola T Foord SM Watson MA Vakkuri O Laitinen JT Important amino acids for the function of the human MT1 melatonin receptor Biochem Pharmacol 2003 65 9 1463 1471 10.1016/S0006-2952(03)00113-8 12732358

[B18] Conway S Canning SJ Barrett P Guardiola-Lemaitre B Delagrange P Morgan PJ The roles of valine 208 and histidine 211 in ligand binding and receptor function of the ovine Mel1a beta melatonin receptor Biochem Biophys Res Commun 1997 239 2 418 423 10.1006/bbrc.1997.7482 9344844

[B19] Conway S Drew JE Mowat ES Barrett P Delagrange P Morgan PJ Chimeric melatonin mt1 and melatonin-related receptors. Identification of domains and residues participating in ligand binding and receptor activation of the melatonin mt1 receptor J Biol Chem 2000 275 27 20602 20609 10.1074/jbc.M002358200 10770942

[B20] Gubitz AK Reppert SM Chimeric and point-mutated receptors reveal that a single glycine residue in transmembrane domain 6 is critical for high affinity melatonin binding Endocrinology 2000 141 3 1236 1244 10.1210/en.141.3.1236 10698201

[B21] Nelson CS Ikeda M Gompf HS Robinson ML Fuchs NK Yoshioka T Neve KA Allen CN Regulation of melatonin 1a receptor signaling and trafficking by asparagine-124 Mol Endocrinol 2001 15 8 1306 1317 10.1210/me.15.8.1306 11463855

[B22] Gerdin MJ Mseeh F Dubocovich ML Mutagenesis studies of the human MT2 melatonin receptor Biochem Pharmacol 2003 66 2 315 320 10.1016/S0006-2952(03)00239-9 12826274

[B23] Mazna P Obsilova V Jelinkova I Balik A Berka K Sovova Z Ettrich R Svoboda P Obsil T Teisinger J Molecular modeling of human MT2 melatonin receptor: the role of Val204, Leu272 and Tyr298 in ligand binding J Neurochem 2004 91 4 836 842 10.1111/j.1471-4159.2004.02758.x 15525337

[B24] Phatnani HP Greenleaf AL Phosphorylation and functions of the RNA polymerase II CTD Genes Dev 2006 20 21 2922 2936 10.1101/gad.1477006 17079683

[B25] Meinhart A Kamenski T Hoeppner S Baumli S Cramer P A structural perspective of CTD function Genes Dev 2005 19 12 1401 1415 10.1101/gad.1318105 15964991

[B26] Goudet G Mugnier S Callebaut I Monget P Phylogenetic Analysis and Identification of Pseudogenes Reveal a Progressive Loss of Zona Pellucida Genes During Evolution of Vertebrates Biol Reprod 2007 10.1095/biolreprod.107.064568 18046012

[B27] Pierre A Gautier M Callebaut I Bontoux M Jeanpierre E Pontarotti P Monget P Atypical structure and phylogenomic evolution of the new eutherian oocyte- and embryo-expressed KHDC1/DPPA5/ECAT1/OOEP gene family Genomics 2007 90 5 583 594 10.1016/j.ygeno.2007.06.003 17913455

[B28] Navarro A Barton NH Chromosomal speciation and molecular divergence--accelerated evolution in rearranged chromosomes Science 2003 300 5617 321 324 10.1126/science.1080600 12690198

[B29] Navajas C Kokkola T Poso A Honka N Gynther J Laitinen JT A rhodopsin-based model for melatonin recognition at its G protein-coupled receptor Eur J Pharmacol 1996 304 1-3 173 183 10.1016/0014-2999(96)00114-8 8813600

[B30] Kimura M The neutral theory of molecular evolution: a review of recent evidence Jpn J Genet 1991 66 4 367 386 10.1266/jjg.66.367 1954033

[B31] Levasseur A Orlando L Bailly X Milinkovitch MC Danchin EG Pontarotti P Conceptual bases for quantifying the role of the environment on gene evolution: the participation of positive selection and neutral evolution Biol Rev Camb Philos Soc 2007 82 4 551 572 10.1111/j.1469-185X.2007.00024.x 17944617

[B32] Ekstrom P Meissl H Evolution of photosensory pineal organs in new light: the fate of neuroendocrine photoreceptors Philos Trans R Soc Lond B Biol Sci 2003 358 1438 1679 1700 10.1098/rstb.2003.1303 14561326 PMC1693265

[B33] Nishio SI Kakizawa T Chatelain G Triqueneaux G Brunet F Rambaud J Lamonerie T Laudet V OTX5 Regulates Pineal Expression of the Zebrafish REV-ERB{alpha} through a New DNA Binding Site Mol Endocrinol 2008 22 1 23 32 10.1210/me.2007-0170 17872382 PMC5419633

[B34] Plouhinec JL Sauka-Spengler T Germot A Le Mentec C Cabana T Harrison G Pieau C Sire JY Veron G Mazan S The mammalian Crx genes are highly divergent representatives of the Otx5 gene family, a gnathostome orthology class of orthodenticle-related homeogenes involved in the differentiation of retinal photoreceptors and circadian entrainment Mol Biol Evol 2003 20 4 513 521 10.1093/molbev/msg085 12654938

[B35] Barrett P Ivanova E Graham ES Ross AW Wilson D Ple H Mercer JG Ebling FJ Schuhler S Dupre SM Loudon A Morgan PJ Photoperiodic regulation of cellular retinoic acid-binding protein 1, GPR50 and nestin in tanycytes of the third ventricle ependymal layer of the Siberian hamster J Endocrinol 2006 191 3 687 698 10.1677/joe.1.06929 17170225

[B36] Ivanova EA Bechtold D Dupre S Brennand J Barrett P Luckman S Loudon AS Altered metabolism in the melatonin-related receptor (GPR50) knock out mouse Am J Physiol Endocrinol Metab 2007 10.1152/ajpendo.00199.2007 17957037

[B37] Bhattacharyya S Luan J Challis B Keogh J Montague C Brennand J Morten J Lowenbeim S Jenkins S Farooqi IS Wareham NJ O'Rahilly S Sequence variants in the melatonin-related receptor gene (GPR50) associate with circulating triglyceride and HDL levels J Lipid Res 2006 47 4 761 766 10.1194/jlr.M500338-JLR200 16436372

[B38] Thomson PA Wray NR Thomson AM Dunbar DR Grassie MA Condie A Walker MT Smith DJ Pulford DJ Muir W Blackwood DH Porteous DJ Sex-specific association between bipolar affective disorder in women and GPR50, an X-linked orphan G protein-coupled receptor Mol Psychiatry 2005 10 5 470 478 10.1038/sj.mp.4001593 15452587

[B39] Drew JE Barrett P Mercer JG Moar KM Canet E Delagrange P Morgan PJ Localization of the melatonin-related receptor in the rodent brain and peripheral tissues J Neuroendocrinol 2001 13 5 453 458 10.1046/j.1365-2826.2001.00651.x 11328456

[B40] Karsch FJ Bittman EL Foster DL Goodman RL Legan SJ Robinson JE Neuroendocrine basis of seasonal reproduction Recent Prog Horm Res 1984 40 185 232 6385166 10.1016/b978-0-12-571140-1.50010-4

[B41] Goldman BD Mammalian photoperiodic system: formal properties and neuroendocrine mechanisms of photoperiodic time measurement J Biol Rhythms 2001 16 4 283 301 10.1177/074873001129001980 11506375

[B42] Malpaux B Neill JD Seasonal regulation of reproduction in mammals. Knobil and Neill's Physiology of Reproduction, Third Edition 2006 3 New York , Elsevier 2231 2281

[B43] Vassilatis DK Hohmann JG Zeng H Li F Ranchalis JE Mortrud MT Brown A Rodriguez SS Weller JR Wright AC Bergmann JE Gaitanaris GA The G protein-coupled receptor repertoires of human and mouse Proc Natl Acad Sci U S A 2003 100 8 4903 4908 10.1073/pnas.0230374100 12679517 PMC153653

[B44] Skinner DC Malpaux B High melatonin concentrations in third ventricular cerebrospinal fluid are not due to Galen vein blood recirculating through the choroid plexus Endocrinology 1999 140 10 4399 4405 10.1210/en.140.10.4399 10499491

[B45] FIGENIX http://www.up.univ-mrs.fr/evol/figenix/

[B46] Gouret P Vitiello V Balandraud N Gilles A Pontarotti P Danchin EG FIGENIX: intelligent automation of genomic annotation: expertise integration in a new software platform BMC Bioinformatics 2005 6 198 10.1186/1471-2105-6-198 16083500 PMC1188056

[B47] Saitou N Nei M The neighbor-joining method: a new method for reconstructing phylogenetic trees Mol Biol Evol 1987 4 4 406 425 3447015 10.1093/oxfordjournals.molbev.a040454

[B48] Felsenstein J Evolutionary trees from DNA sequences: a maximum likelihood approach J Mol Evol 1981 17 6 368 376 10.1007/BF01734359 7288891

[B49] Dayhoff MO Schwartz RM Orcutt BC NBR F A model of evolutionary change in proteins Atlas of Protein Sequence and Structure 1978 Washington D.C. 345 352

[B50] Thompson JD Higgins DG Gibson TJ CLUSTAL W: improving the sensitivity of progressive multiple sequence alignment through sequence weighting, position-specific gap penalties and weight matrix choice Nucleic Acids Res 1994 22 22 4673 4680 10.1093/nar/22.22.4673 7984417 PMC308517

[B51] Birney E Clamp M Durbin R GeneWise and Genomewise Genome Res 2004 14 5 988 995 10.1101/gr.1865504 15123596 PMC479130

[B52] Yang Z PAML: a program package for phylogenetic analysis by maximum likelihood Comput Appl Biosci 1997 13 5 555 556 9367129 10.1093/bioinformatics/13.5.555

[B53] Zhang J Nielsen R Yang Z Evaluation of an improved branch-site likelihood method for detecting positive selection at the molecular level Mol Biol Evol 2005 22 12 2472 2479 10.1093/molbev/msi237 16107592

[B54] ENSEMBL http://www.ensembl.org/Gallus_gallus/syntenyview?otherspecies=Homo_sapiens;chr=4

[B55] TBLASTN http://www.ncbi.nlm.nih.gov/BLAST/

[B56] EMBL http://www.ebi.ac.uk/Tools/clustalw/index.html

[B57] EMBOSS http://bioweb.pasteur.fr/seqanal/interfaces/water.html

[B58] BLAT http://genome.brc.mcw.edu/

[B59] Altschul SF Madden TL Schaffer AA Zhang J Zhang Z Miller W Lipman DJ Gapped BLAST and PSI-BLAST: a new generation of protein database search programs Nucleic Acids Res 1997 25 17 3389 3402 10.1093/nar/25.17.3389 9254694 PMC146917

[B60] Callebaut I Labesse G Durand P Poupon A Canard L Chomilier J Henrissat B Mornon JP Deciphering protein sequence information through hydrophobic cluster analysis (HCA): current status and perspectives Cell Mol Life Sci 1997 53 8 621 645 10.1007/s000180050082 9351466 PMC11147222

[B61] Eudes R Le Tuan K Delettre J Mornon JP Callebaut I A generalized analysis of hydrophobic and loop clusters within globular protein sequences BMC Struct Biol 2007 7 2 10.1186/1472-6807-7-2 17210072 PMC1774571

[B62] Katoh K Kuma K Toh H Miyata T MAFFT version 5: improvement in accuracy of multiple sequence alignment Nucleic Acids Res 2005 33 2 511 518 10.1093/nar/gki198 15661851 PMC548345

[B63] Rivara S Lorenzi S Mor M Plazzi PV Spadoni G Bedini A Tarzia G Analysis of structure-activity relationships for MT2 selective antagonists by melatonin MT1 and MT2 receptor models J Med Chem 2005 48 12 4049 4060 10.1021/jm048956y 15943478

[B64] Marti-Renom MA Stuart AC Fiser A Sanchez R Melo F Sali A Comparative protein structure modeling of genes and genomes Annu Rev Biophys Biomol Struct 2000 29 291 325 10.1146/annurev.biophys.29.1.291 10940251

[B65] Laskowski RA Moss DS Thornton JM Main-chain bond lengths and bond angles in protein structures J Mol Biol 1993 231 4 1049 1067 10.1006/jmbi.1993.1351 8515464

