# Correction: GPR50 is the mammalian ortholog of Mel1c: Evidence of rapid evolution in mammals

**DOI:** 10.1186/1471-2148-12-28

**Published:** 2012-03-07

**Authors:** Laurence Dufourny, Anthony Levasseur, Martine Migaud, Isabelle Callebaut, Pierre Pontarotti, Benoit Malpaux, Philippe Monget

**Affiliations:** 1 Physiologie de la Reproduction et des Comportements, UMR 6175 INRA-CNRS-Université François Rabelais de Tours-Haras Nationaux, 37380 Nouzilly, France; 2 EA3781: Evolution Biologique - Laboratoire de PhyloGénomique - Université de Provence - Marseille - France; 3 Biologie Structurale, IMPMC, UMR7590, CNRS, Université Paris 6 et 7, 75015 Paris, France

## Correction

The core of the text as well as the legends remain the same as in our article [1] since the changes provided here do not change any of the outcomes of the former study including the results and our related conclusions. However, new figure files are provided since some arrows and stars have been previously misplaced in figures three A (figure 1) and four (figure 2), and the former figure six (figure 3) showed the GPR50 mouse sequence instead of the human sequence as stated in the legend [1].

**Figure 1:**
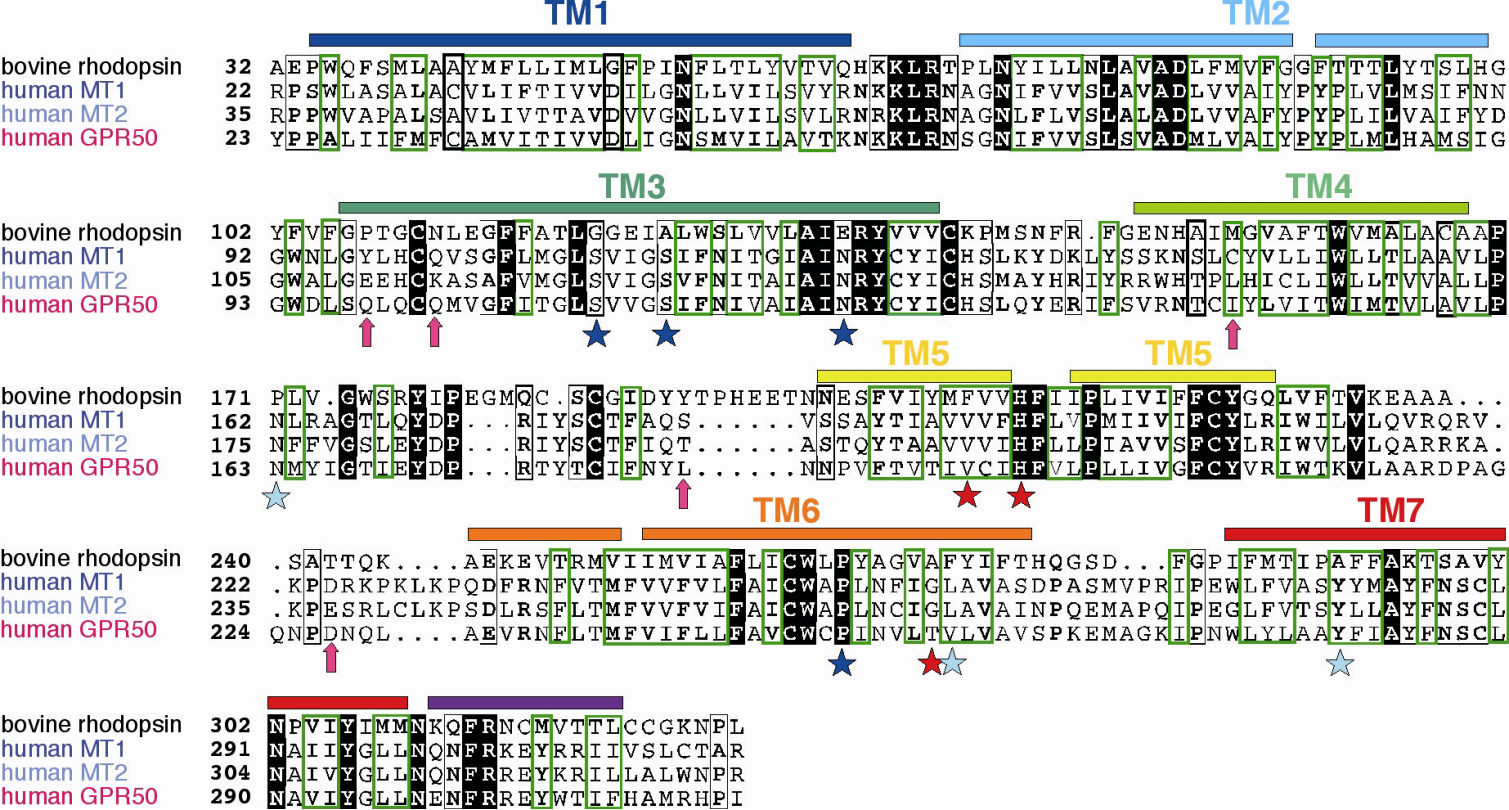
**Phylogenetic analysis of the GPR50/MT1/MT2/Mel1c genes**. (A) Overall phylogenetic tree showing 3 groups of genes: GPR50, MT1/MT2, and Mel1c genes and the animal orders where each branch is expressed. The trees (npl) are the fusion of three phylogenetic trees built based on Neighbour joining, maximum Parsimony and maximum Likelihood (see "Materials and Methods" section for further details). The italic letters correspond to the name given to the branches for the likelihood ratio tests (B) Phylogenetic tree of GPR50 genes. Please note that only mammalian species appear in the tree. (C) Phylogenetic tree of MT1 genes (D) Phylogenetic tree of MT2 genes. (E) Phylogenetic tree of Mel1c genes that do seem to appear only in non mammalian species. Bootstrap values are reported for each npl method.

**Figure 2:**
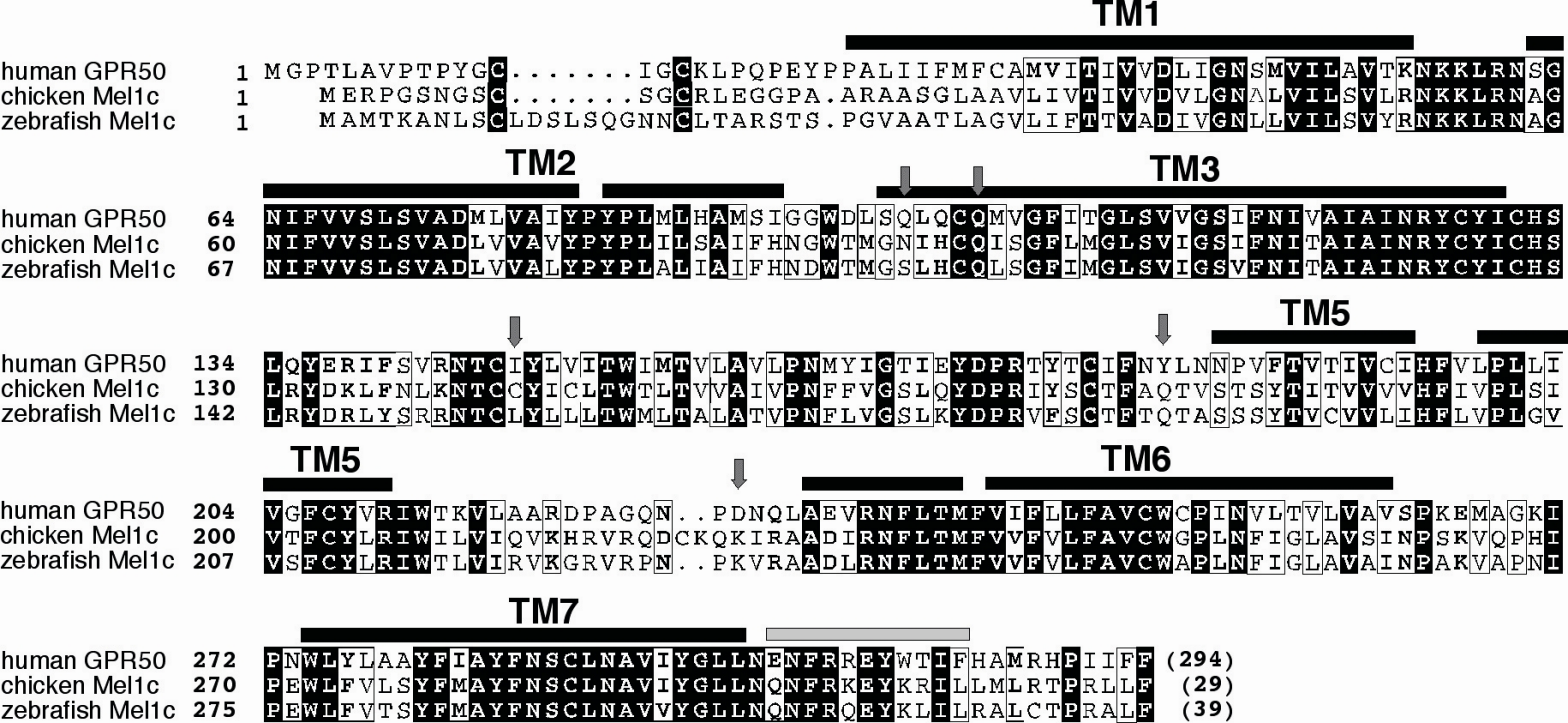
**Synteny of Mel1c/GPR50 genes and neighbours in vertebrate genomes**. Note that genes are found on chromosome 5 in zebra fish and on chromosome 4 in chicken while they are found on chromosome X in other depicted species. Please note that synteny is mostly conserved for bHLHPAS, 2610030H06 RIK, Mel1c, HMG2A, CD99, and myotubularin related protein in opossum and mammalian species despite the integration of new genes coding for hypothetical proteins (opossum, chimpanzee, cow), ribosomal proteins (dog, chimpanzee, man), NGFI-A binding protein (chimpanzee, man), Utbf (mouse) and MAGE (cattle) proteins. It is also of note that several genes surrounding Mel1c in zebra fish (pdcd8, nono, and the two hypothetical proteins) present high identities with genes found on chromosome X in mouse but not in the GPR50 locus (unpublished data). p.d.: predicted gene. Chrm: chromosome.

**Figure 3:**
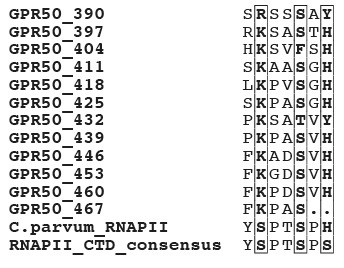
**Sequence alignment of human MT1, MT2 and GPR50 with bovine rhodopsin **(pdb 1F88). Sequence identities are reported white on a black background, whereas sequence similarities are boxed (A). The positions of the transmembrane helices, as observed in the bovine rhodopsin structure, are reported above its sequence. Arrows indicate the positions of the amino acids that, in GPR50, evolved under positive selection. Stars indicate amino acids which have been shown to play a key role for melatonin binding in MT1 (dark blue), MT2 (light blue) or both (red). A ribbon representation of the GPR50 3D structure model is represented (B), with transmembrane helices colored according to the sequence alignment. Amino acids evolving under positive selection and amino acids important for melatonin binding in MT1/MT2 are shown according to the colors reported in the sequence alignment.

## References

[B1] Dufourny L Levasseur A Migaud M Callebaut I Pontarotti P Malpaux B Monget P GPR50 is the mammalian ortholog of Mel1c: evidence of rapid evolution in mammals BMC Evol Biol 2008 8 105 10.1186/1471-2148-8-105 18400093 PMC2323367

