# Supplementary figures and images for: GPR50 is the mammalian ortholog of Mel1c: Evidence of rapid evolution in mammals
Source: BMC Evol Biol. 2008 Apr 9;8:105. doi: 10.1186/1471-2148-8-105 (PMC2323367; doi:10.1186/1471-2148-8-105)

# RIK2610030H06

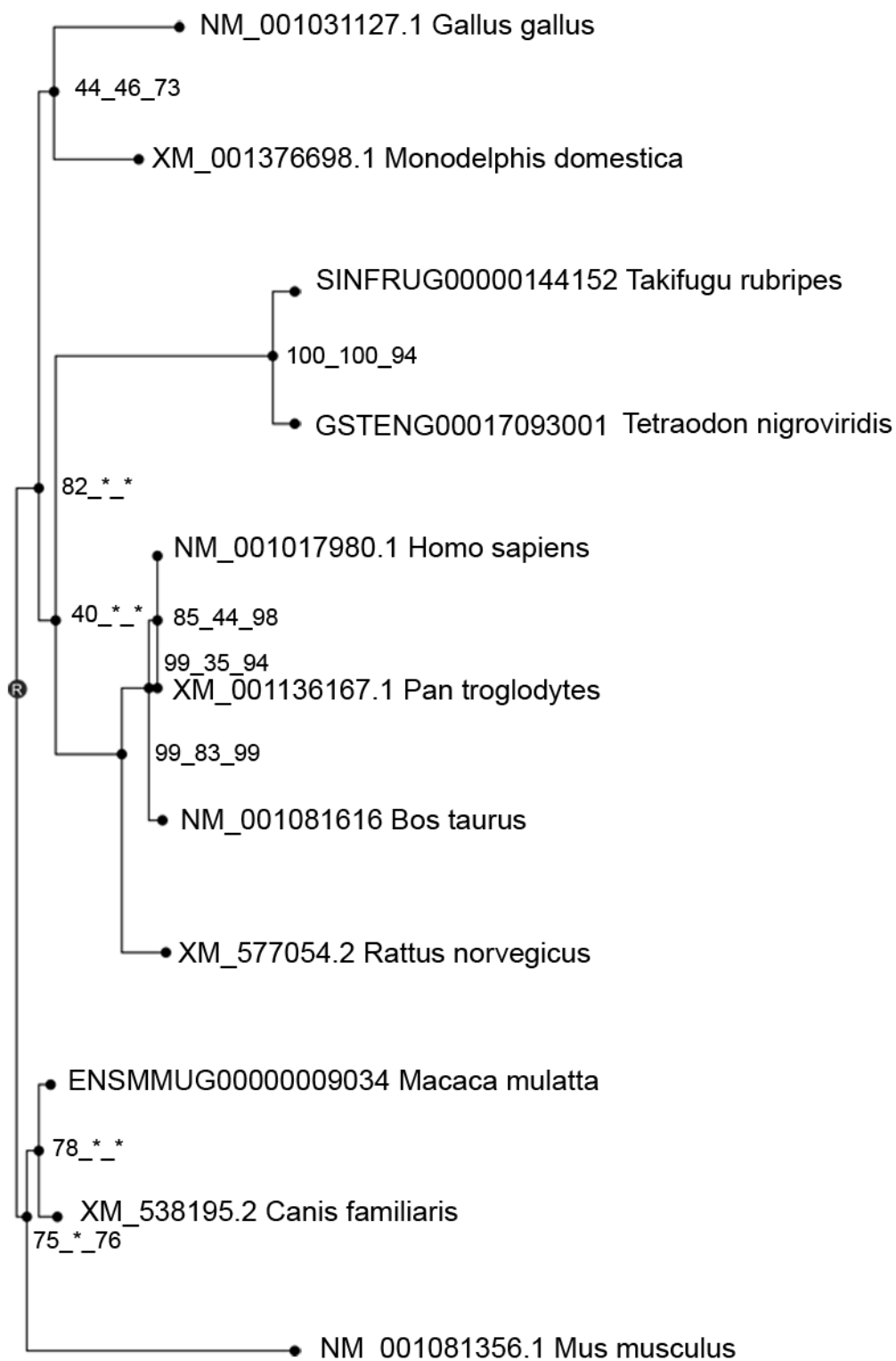

Supplement: Additional file 1 — Phylogenetic tree for 2610030H06Rik gene. The figure provided shows the odd organization of species within the tree (for example, chicken associated in the same branch than opossum), which suggests a fast evolution of the 2610030H06Rik gene. [file 1471-2148-8-105-S1.pdf]

## HMG2A

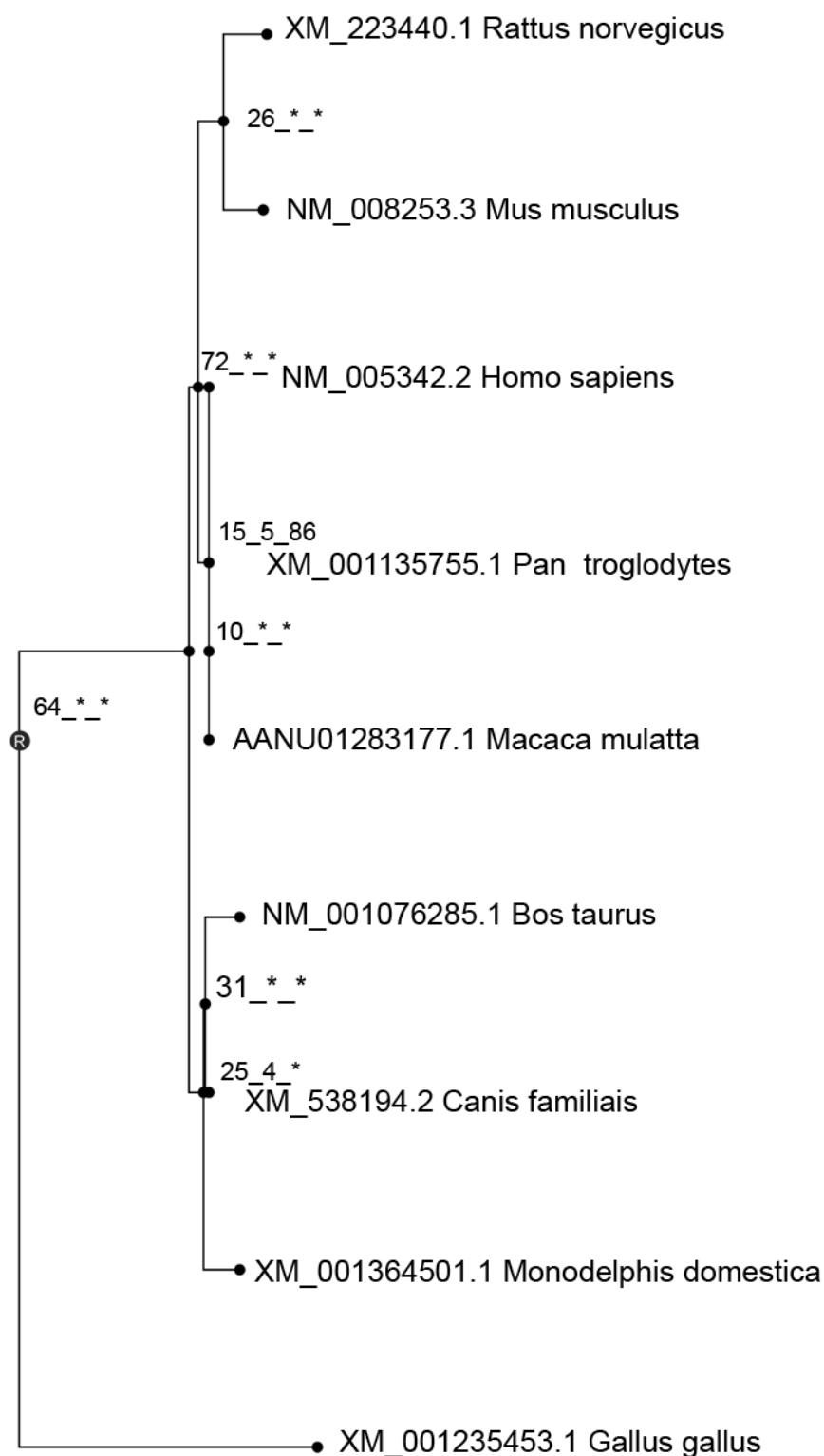

Supplement: Additional file 2 — Phylogenetic tree for HMG2A gene. The figure provided highlights the poor bootstrap values in the tree which suggests a fast evolution of HMG2A gene. [file 1471-2148-8-105-S2.pdf]
